# High-performance automated abstract screening with large language model ensembles

**DOI:** 10.1093/jamia/ocaf050

**Published:** 2025-03-22

**Authors:** Rohan Sanghera, Arun James Thirunavukarasu, Marc El Khoury, Jessica O’Logbon, Yuqing Chen, Archie Watt, Mustafa Mahmood, Hamid Butt, George Nishimura, Andrew A S Soltan

**Affiliations:** Oxford University Hospitals NHS Foundation Trust, Oxford OX3 9DU, United Kingdom; Oxford University Clinical Academic Graduate School, Medical Sciences Division, University of Oxford, Oxford OX3 9DU, United Kingdom; Oxford University Hospitals NHS Foundation Trust, Oxford OX3 9DU, United Kingdom; Nuffield Department of Clinical Neurosciences, Medical Sciences Division, University of Oxford, Oxford OX3 9DU, United Kingdom; School of Clinical Medicine, University of Cambridge, Cambridge CB2 0SP, United Kingdom; Georgetown University School of Medicine, Georgetown University, Washington, DC 20007, United States; MedStar Washington Hospital Center, Washington, DC 20010, United States; GKT School of Medical Education, King’s College London, London WC2R 2LS, United Kingdom; School of Clinical Medicine, University of Cambridge, Cambridge CB2 0SP, United Kingdom; Oxford Medical School, Medical Sciences Division, University of Oxford, Oxford OX3 9DU, United Kingdom; UCL Medical School, University College London, London WC1E 6DE, United Kingdom; School of Clinical Medicine, University of Cambridge, Cambridge CB2 0SP, United Kingdom; School of Clinical Medicine, University of Cambridge, Cambridge CB2 0SP, United Kingdom; Oxford University Hospitals NHS Foundation Trust, Oxford OX3 9DU, United Kingdom; Department of Oncology, Medical Sciences Division, University of Oxford, Oxford OX3 7DQ, United Kingdom

**Keywords:** artificial intelligence, large language model, systematic review, abstract screening, foundation model, evidence synthesis

## Abstract

**Objective:**

Abstract screening is a labor-intensive component of systematic review involving repetitive application of inclusion and exclusion criteria on a large volume of studies. We aimed to validate large language models (LLMs) used to automate abstract screening.

**Materials and Methods:**

LLMs (GPT-3.5 Turbo, GPT-4 Turbo, GPT-4o, Llama 3 70B, Gemini 1.5 Pro, and Claude Sonnet 3.5) were trialed across 23 Cochrane Library systematic reviews to evaluate their accuracy in zero-shot binary classification for abstract screening. Initial evaluation on a balanced development dataset (n = 800) identified optimal prompting strategies, and the best performing LLM-prompt combinations were then validated on a comprehensive dataset of replicated search results (n = 119 695).

**Results:**

On the development dataset, LLMs exhibited superior performance to human researchers in terms of sensitivity (LLM_max_ = 1.000, human_max_ = 0.775), precision (LLM_max_ = 0.927, human_max_ = 0.911), and balanced accuracy (LLM_max_ = 0.904, human_max_ = 0.865). When evaluated on the comprehensive dataset, the best performing LLM-prompt combinations exhibited consistent sensitivity (range 0.756-1.000) but diminished precision (range 0.004-0.096) due to class imbalance. In addition, 66 LLM-human and LLM-LLM ensembles exhibited perfect sensitivity with a maximal precision of 0.458 with the development dataset, decreasing to 0.1450 over the comprehensive dataset; but conferring workload reductions ranging between 37.55% and 99.11%.

**Discussion:**

Automated abstract screening can reduce the screening workload in systematic review while maintaining quality. Performance variation between reviews highlights the importance of domain-specific validation before autonomous deployment. LLM-human ensembles can achieve similar benefits while maintaining human oversight over all records.

**Conclusion:**

LLMs may reduce the human labor cost of systematic review with maintained or improved accuracy, thereby increasing the efficiency and quality of evidence synthesis.

## Background and significance

Systematic review underpins evidence-based medicine (EBM) as the primary method for synthesizing data from previously reported clinical studies^1^^,^^2^ as well as knowledge from research in non-medical fields.^1^^,^^3^^,^^4^ Good practices include transparent reporting and reproducible methodology, and checklists and guidance exist to support adherence to accepted standards of conduct and reporting.^1^^,^^5^ Some tasks involved in the systematic review process can be labor-intensive, repetitive, and text-based with formulaic and algorithmic schema used to maximize reproducibility.^6^ Examples include trialing search strategies, screening abstracts and full texts for inclusion, and extracting data from included studies.^3^^,^^7^

Abstract screening is the process of selecting articles identified by the search strategy that meet pre-specified criteria for inclusion and is typically performed by 2 or more researchers with domain-specific expertise. Screeners use the title and abstract of each record to determine eligibility and make decisions to include or exclude accordingly. Tools to streamline abstract screening are already in wide use but researchers using these tools are limited to a maximum rate of screening of around 2 abstracts per minute.^8^^,^^9^ The use of emerging artificial intelligence (AI) applications has been posited as a means of improving the accuracy and efficiency of abstract screening.^10^^,^^11^

Computational natural language processing has advanced significantly with the development and deployment of large language models (LLMs).^12^ LLMs are pretrained on large volumes of human-produced text and then instruction-tuned on a wide variety of tasks to develop remarkable abilities to interpret and generate text in multiple languages.^12^ In medicine, LLMs have garnered significant attention for attaining comparable results to clinicians in examinations and other reasoning tasks, but are yet to be deployed in a decision-making capacity in real-world settings.^13^^,^^14^ Healthcare research offers an arena in which LLMs may be deployed with less direct risk to patients, and automating systematic review is one such avenue of research.^15^ However, high accuracy is critical to ensure that the conclusions drawn are valid, as systematic reviews are the highest weighted evidence when designing treatment algorithms and providing advice to clinicians and patients.^7^^,^^16^ As a reasoning and binary classification task, abstract screening is amenable to automation using LLMs.

## Objectives

We aimed to provide a general estimate of the abstract screening performance of a variety of LLMs; demonstrate an effective workflow to optimize accuracy and sensitivity of automated abstract screening; and show how LLMs and human researchers may be combined to maximize efficiency and accuracy. We approached abstract screening as a zero-shot binary classification problem, thereby maximizing generalizability to other systematic reviews and literature syntheses by limiting the requirement for domain-specific fine-tuning or prompt engineering.

## Materials and methods

### LLM pipelines for automated abstract screening

LLM screening was undertaken using pipelines implementing application programming interface (APIs) for open- and closed-source LLMs. GPT-3.5 Turbo (gpt-3.5-turbo-0125), GPT-4 Turbo (gpt-4-0125-preview), and GPT-4o (gpt-4o-2024-05-13) models were accessed through the Azure OpenAI Service, using the OpenAI (1.23.2) Python package. Llama 3 70B (meta-llama-3-70b) was hosted on Replicate.com; Claude Sonnet 3.5 (claude-3-5-sonnet@20240620), and Gemini 1.5 Pro (gemini-1.5-pro-001) models were accessed through Google Cloud’s Vertex AI platform.

### Data selection and preparation

All systematic reviews from the latest issue of the Cochrane Database of Systematic Reviews at the time of protocol development (2023, Issue 8) were used for experiments.^17–39^ The Cochrane Library was selected for its gold standard methodology, consistency in reporting (including search strategies and inclusion criteria), and unbiased coverage of topics across medicine and surgery. For each of 23 reviews in Issue 8 (2023), the original search strategy was replicated using identical keyword combinations on the databases specified in the reviews’ appendices. Replicated searches often returned different numbers of records to those reported in the original reviews, likely due to a combination of use of sources other than databases, changes to records stored within databases, as well as errors in search strategy reporting.^40^ To ensure temporal consistency, records published after the date of search listed in the reviews were excluded from subsequent analyses. The inclusion lists of each review were used as the ground truth: gold standard examples of articles which should have been included on the basis of the reviews’ protocols.

The initial corpus comprised 128 299 articles from replicated and de-duplicated searches across all 23 systematic reviews. During data cleaning, 8604 articles (6.71%) with missing abstracts were excluded from further analysis, resulting in a final dataset of 119 695 articles. We structured our evaluation using 2 datasets:

1. A comprehensive dataset of all 119 695 articles, which was used for final evaluation of optimal LLM-prompt combinations, as a faithful reflection of the real-world task of abstract screening.
2. A balanced development dataset of 800 articles, generated using articles from the inclusion lists and a random sample of 23 excluded articles from each review. To maintain computational feasibility, this subset was used to systematically develop and evaluate a range of prompts with varying inclusion thresholds, characterizing the impact of prompt design on model performance.

### Prompt development

Multiple prompts were developed to investigate the effect of prompt engineering on abstract screening performance. A generalized prompt structure was initially developed through exploratory analysis on a limited number of articles using GPT-3.5, providing an unbiased description of the abstract screening task (“none”). This default prompt was then systematically iterated and evaluated on the development dataset (n = 800) by adjusting the threshold for inclusion, producing a spectrum of prompts with progressively higher bias towards inclusion: “mild,” “moderate,” “heavy,” and “extreme.” A control prompt, “title,” was also tested, in which only the title of each record was presented. The prompt for Llama 3 was subtly adjusted to incorporate special tokens and align with its specific prompt structure. The final wording of each prompt is provided in Supplementary Material S1.

For each systematic review, prompts were constructed using the task description described above, review’s title, and inclusion criteria. Based on preliminary testing, model parameters were adjusted to produce deterministic, concise outputs suitable for abstract screening: “temperature” and “max_tokens” parameters were specified as 0.2 and 5, respectively. Exploratory testing was performed for the “frequency_penalty” and “presence_penalty” parameters using Llama 3 70B, but no significant improvements in performance were yielded (Supplementary Material S2). We, therefore, retained default values for those parameters in subsequent deployment. In cases where the API returned an error or an invalid decision, queries were repeated with exponential backoff to address rate-liming.

Where models repeatedly returned an error message, content violation note, or no interpretable output, a label of “include” was assigned to avoid excluding potentially eligible records before full text screening. This schema aimed to maximize model sensitivity even at the expense of overall accuracy, as false negatives (exclusion of eligible records) are a more damaging error than false positives (including ineligible records for full text screening); because eligible records that are excluded will not be used in subsequent evidence synthesis.

### Evaluation methodology

Validation of LLMs was performed in 2 phases. Initial evaluation was conducted on the development dataset to make systematic comparisons between LLM-prompt combinations. In these trials, each LLM-prompt combination was applied 3 times over the development dataset to establish consistency. As a comparator, 3 human researchers independently screened the same abstracts in the development dataset.

For extended validation, the best-performing LLM-prompt combinations for each model were evaluated on the comprehensive dataset to assess real-world performance. Prompt selection for full dataset evaluation was based on balanced accuracy—the arithmetic mean of sensitivity and specificity. An exception was made for GPT-3.5, where the “heavy” bias prompt was selected despite not exhibiting optimal balanced accuracy, because it achieved perfect sensitivity which would be most compatible with autonomous deployment in real-world screening. GPT-4 was excluded from full dataset evaluation as its successor, GPT-4o, demonstrated superior performance, efficiency, and cost.

Because independent replication of abstract screening by human researchers across the comprehensive dataset was not feasible, comparisons were made with statistics derived from the original Cochrane Library reviews. Statistics were calculated using the reported numbers of records screened and excluded after abstract screening. Sensitivity was considered 100% for the original authors, because their inclusion decisions were used as the ground truth to determine which records were eligible for inclusion.

### Performance metrics

Performance was evaluated using confusion matrices with the following definitions applied:

- True positive: eligible record correctly included
- True negative: ineligible record correctly excluded
- False positive: ineligible record incorrectly included
- False negative: eligible record incorrectly excluded

Sensitivity, specificity, and accuracy calculations were subsequently performed to provide interpretable measures of abstract screening performance. For one review where no eligible records were included, sensitivity was represented as 100% rather than indeterminate to facilitate quantitative comparisons.^36^ Given the relative scarcity of included articles in systematic review, precision (positive predictive value) and recall (sensitivity) were selected as primary outcome measures, as these provide more informative comparisons than sensitivity and specificity for imbalanced binary classification tasks.^41^

For the development dataset, Kappa statistics were calculated for repeat trials of each LLM-prompt combination and between human screeners, to quantify screening consistency. Correlation analysis between human and LLM performance was undertaken to explore whether specific reviews were consistently more challenging across both human and LLM screeners, with coefficients of determination (R^2^) calculated to assess the strength of these relationships.

### Implementation simulation

LLM and human screening decisions were combined in 6 distinct ensembled configurations to explore potential deployment strategies for automated abstract screening. All possible combinations *in parallel* and *in series* were tested. For in parallel ensembles, a single “include” decision from either component was sufficient for article inclusion, whereas for in series ensembles both components had to reach an “include” decision for the article to be included. For the development dataset, all LLMs, prompts, and human researchers were combined in every configuration. For each ensemble, precision and recall were calculated to identify optimal combinations in terms of precision and recall. The highest performance ensembles were compared against original Cochrane review performance metrics to assess relative effectiveness.

Finally, to illustrate the potential efficiency gain of automated abstract screening, ensembles of optimal LLM-prompt combinations were trialed across the comprehensive dataset. Workload reduction was calculated as the number of correctly excluded articles per 100 screened records; this corresponds to the proportion of articles that human reviewers would not need to appraise subsequently, as all included abstracts would be reappraised at the full-text screening stage.

### Technical details

All experiments were conducted in Python (Python Software Foundation, Wilmington, Delaware, USA; version 3.11.5). Data analysis and visualization were conducted in R (R Foundation for Statistical Computing, Vienna, Austria; version 4.2.1) and Affinity Designer (Serif Europe Ltd, West Bridgford, UK; version 1.10.6). All code required to replicate experiments and analysis is hosted on GitHub (https://github.com/RohanSanghera/GEN-SYS).

## Results

A total of 23 systematic reviews were used for experiments, comprising the entirety of 2023 Issue 8 of the *Cochrane Database of Systematic Reviews* (Table 1).^17–39^ These reviews exhibited a wide range of specialties, interventions, and sizes in terms of included studies and participants. Two reviews featured lead authors with the same name and were referred to as Singh-1^34^ and Singh-2^33^ to distinguish between them.

**Table 1.** Characteristics of 23 systematic reviews taken from 2023 Issue 8 of the Cochrane Database of Systematic Reviews, used for all experiments.

Lead author	Title	n (search results)	n (included records)
Bellon	Perioperative glycaemic control for people with diabetes undergoing surgery	3693	23
Buchan	Medically assisted hydration for adults receiving palliative care	5043	4
Clezar	Pharmacological interventions for asymptomatic carotid stenosis	6476	31
Cutting	Intracytoplasmic sperm injection versus conventional in vitro fertilisation in couples with males presenting with normal total sperm count and motility	3092	3
Dopper	High flow nasal cannula for respiratory support in term infants	8768	8
Ghoraba	Pars plana vitrectomy with internal limiting membrane flap versus pars plana vitrectomy with conventional internal limiting membrane peeling for large macular hole	2690	5
Hjetland	Vocabulary interventions for second language (L2) learners up to six years of age	6238	12
Karkou	Dance movement therapy for dementia	706	3
Lin	Hyperbaric oxygen therapy for late radiation tissue injury	773	16
Lynch	Interventions for the uptake of evidence‐based recommendations in acute stroke settings	20319	7
Malik	Fibrin‐based haemostatic agents for reducing blood loss in adult liver resection	3685	20
Mohamed	Prostaglandins for adult liver transplanted recipients	304	11
Roy	Interventions for chronic kidney disease in people with sickle cell disease	5891	28
Santos	Prophylactic anticoagulants for non‐hospitalised people with COVID‐19	17 380	5
Setthawong	Extracorporeal shock wave lithotripsy (ESWL) versus percutaneous nephrolithotomy (PCNL) or retrograde intrarenal surgery (RIRS) for kidney stones	1880	21
Sévaux	Paracetamol (acetaminophen) or non‐steroidal anti‐inflammatory drugs, alone or combined, for pain relief in acute otitis media in children	10 826	4
Singh-1	Blue‐light filtering spectacle lenses for visual performance, sleep, and macular health in adults	195	17
Singh-2	Interventions for bullous pemphigoid	312	16
Sulewski	Topical ophthalmic anesthetics for corneal abrasions	7016	9
Sulistyo	Enteral tube feeding for amyotrophic lateral sclerosis/motor neuron disease	189	0
White	Oxygenation during the apnoeic phase preceding intubation in adults in prehospital, emergency department, intensive care and operating theatre environments	13 549	22
Younis	Hydrogel dressings for donor sites of split‐thickness skin grafts	425	2
Zhu	Expanded polytetrafluoroethylene (ePTFE)‐covered stents versus bare stents for transjugular intrahepatic portosystemic shunt in people with liver cirrhosis	246	4

The reviews covered a broad range of specialties, interventions, sample sizes (in terms of studies and participants), and methodologies (meta-analyses and narrative syntheses). Numbers of records and included studies are based on replicated searches undertaken for experimental purposes, rather than the numbers reported in the original reviews.

### Prompt design determines LLM screening behavior

**Figure 1.**
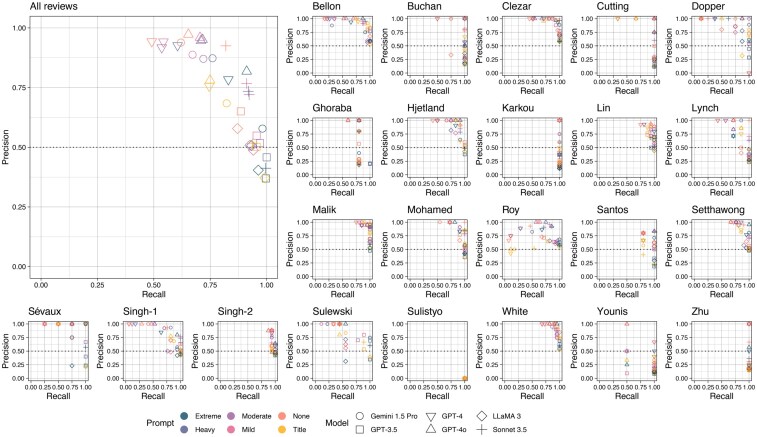
Precision (positive predictive value) and recall (sensitivity) of 6 LLMs tasked with automated abstract screening on the development dataset (n = 800), across a range of 6 prompts with varying bias towards inclusion. Sensitivity was deemed 100% for all models working with Sulistyo et al,^36^ as there were no articles deemed eligible for inclusion in the original review. Performance was highly variable between models and across different prompts but was comparable to human researchers conducting the same abstract screening task. When used with a prompt containing a “heavy” bias towards inclusion, GPT-3.5 exhibited perfect (100%) sensitivity across every review, meaning that all eligible articles were correctly included. For other models, the optimal prompt taken forward in further experiments was determined by the highest calculated balanced accuracy: “none” (no bias towards inclusion) for Llama 3 and Sonnet, “heavy” bias for Gemini Pro, and “extreme” bias for GPT-4o. Balanced accuracy was optimal for GPT-4 with the “extreme” bias prompt, but was inferior to its successor model, GPT-4o, in addition to exhibiting worse efficiency.

Initial evaluation of LLM performance was conducted on a balanced development dataset of 800 records, to facilitate systematic comparison of prompt engineering strategies. Each LLM-prompt combination was tested across all reviews, with performance varying substantially with respect to prompt design (Figure 1). There was a clear trade-off between recall (sensitivity) and precision (positive predictive value): as recall increased, precision tended to decrease (Pearson’s correlation coefficient, R = −0.47, 95% confidence interval −0.53 to −0.42, *P* < .001), in-keeping with a lower threshold for inclusion. Recall varied significantly as the prompt was changed, consistent with prompt design being responsible for the difference in threshold for inclusion (Kruskal-Wallis test, χ^2^ = 62.5, *P* < .001). Moreover, differences were directed in the same manner as the language used in the prompt: a heavier bias towards inclusion resulted in more records being included.

### LLM can match or exceed human screening performance on a balanced dataset

The performance of human researchers replicating abstract screening over the development dataset lay within the range of accuracy, sensitivity, and specificity of LLMs tasked with screening the same abstracts (Table 2). For every calculated performance metric, an LLM (GPT-3.5, GPT-4, or Sonnet) exhibited the strongest performance; higher than all 3 human researchers. Similar comparative performance was observed when results were stratified by review (Supplementary Material S2).

**Table 2.** Performance of 3 human researchers (Alpha, Bravo, and Charlie) and 6 LLMs (GPT-3.5 Turbo, GPT-4 Turbo, GPT-4o, Gemini 1.5 Pro, Llama 3 70B, and Claude Sonnet 3.5.

Human/Model	Optimal prompt	Sensitivity (recall)	Specificity	Balanced accuracy	Precision (PPV)	NPV	F1-score
Alpha	N/A	0.745	0.962	0.854	0.910	0.881	0.819
Bravo	N/A	0.720	0.964	0.842	0.911	0.870	0.804
Charlie	N/A	0.775	0.955	0.865	0.897	0.892	0.832
GPT-3.5	Heavy	1.000	0.393	0.697	0.458	1.000	0.628
GPT-4	Extreme	0.605	0.975	0.857	0.927	0.828	0.732
GPT-4o	Extreme	0.911	0.896	0.904	0.818	0.952	0.862
Gemini 1.5 Pro	Heavy	0.760	0.943	0.852	0.873	0.885	0.813
LLaMA 3	None	0.871	0.675	0.773	0.578	0.911	0.695
Sonnet 3.5	None	0.819	0.966	0.893	0.925	0.913	0.869

All used with their respective optimal prompts, replicating abstract screening over the development dataset (n = 800). LLMs (specifically GPT-3.5, GPT-4, and Sonnet) exhibited the highest performance in terms of every measured metric. N/A = not applicable.

The consistency of LLM decisions was evaluated through repeat screening trials on the development dataset for each LLM-prompt combination. LLMs exhibited high internal consistency, with Kappa statistics varying between reviews (Table S1). Where the optimal prompt was used, κ_GPT-3.5_ ranged between 0.487 and 1.000 (median = 0.868), κ_GPT-4_ between 0.870 and 1.000 (median = 0.957), κ_GPT-4o_ between 0.787 and 1.000 (median = 0.941), κ_Gemini Pro_ between 0.927 and 1.000 (median = 1.000), κ_Llama 3_ between 0.642 and 1.000 (median = 0.881), and κ_Sonnet 3.5_ between 0.903 and 1.000 (median = 1.000). Human researchers screening the same abstracts exhibited more inconsistency than the LLMs but similar variation across reviews, with a median Kappa statistic of 0.827 (range −0.045 to 1.000).

**Figure 2.**
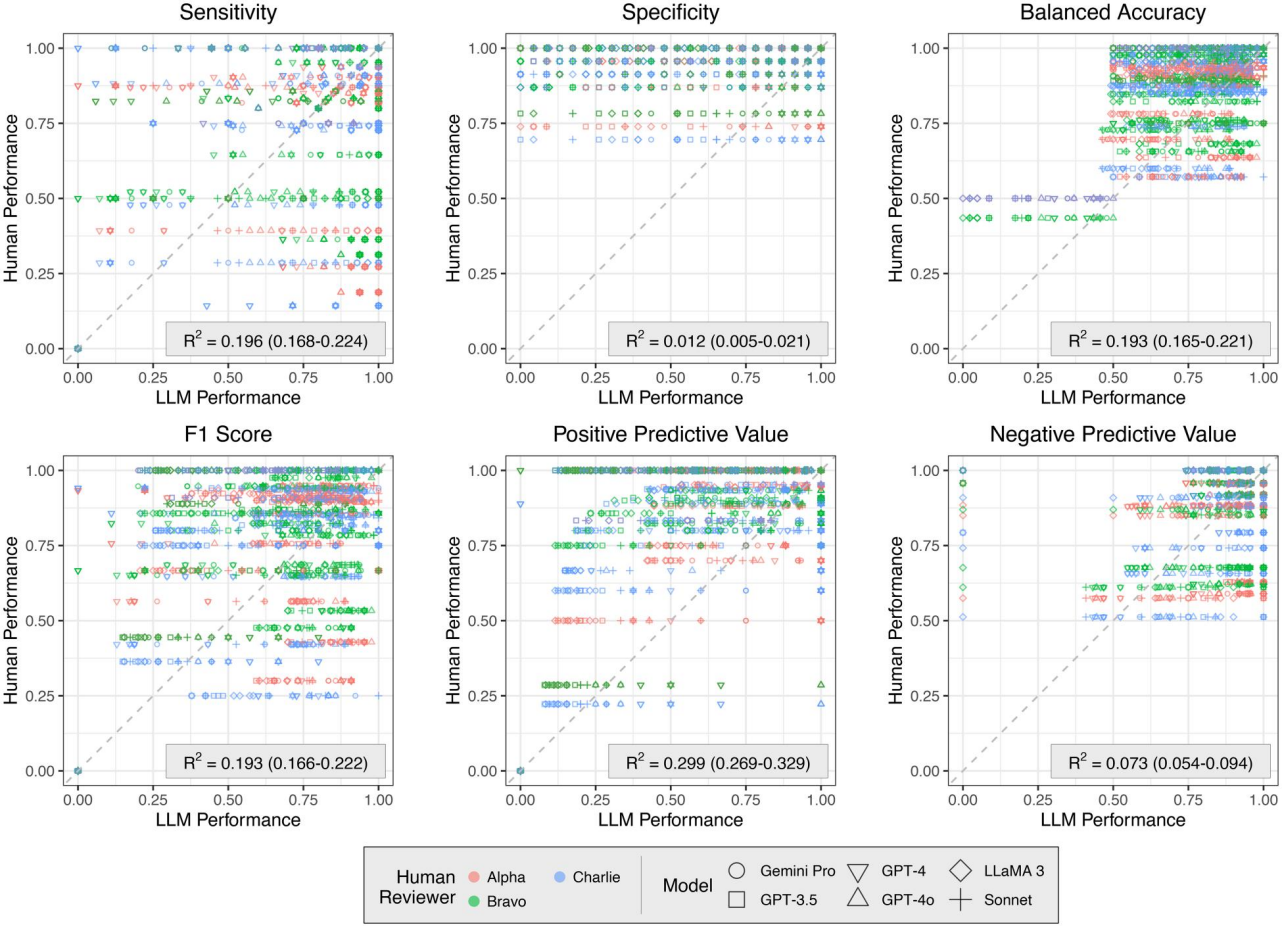
Correlational analysis, undertaken on results obtained from the development dataset, to investigate whether review-centric factors influenced the abstract screening performance of LLMs and human researchers replicating the work of Cochrane systematic review authors. Between 1.2% and 29.9% of the variation in human performance was predictable based on LLM performance, with higher coefficients of determination for sensitivity, F1-score, balanced accuracy, and positive predictive value. It is likely that review-centric factors—such as clarity and comprehensiveness of reporting—contribute to this relationship. Less association was observed for sensitivity and negative predictive value, perhaps in part due to less overall variation in human and LLM performance when measured with those metrics.

Correlational analysis was undertaken to explore whether review-centric factors were likely to determine observed variation in agreement and performance across different systematic reviews. Despite noise generated by dependency of performance on the model or human evaluator, and prompt used with the LLMs, a consistent association between human and LLM performance was observed across the Cochrane reviews (Figure 2). Consistent association between human performance and LLM performance was determined by calculation of the coefficient of determination. Positive association was greatest for sensitivity (R^2^ = 0.196), balanced accuracy (R^2^ = -.193), F1-score (R^2^ = 0.193), and positive predictive value (R^2^ = 0.299); and lower for negative predictive value (R^2^ = 0.073) and specificity (R^2^ = 0.012). These results indicated that review-centric factors—such as clarity and comprehensiveness of reporting—may affect abstract screening performance in addition to LLM and human factors such as intrinsic aptitude, expertise, and prompt engineering.

### LLMs maintain high sensitivity when extended to a real-world dataset

LLM precision decreased markedly when evaluated on the comprehensive dataset (n = 119 695), relative to the balanced development dataset (Table 3). This expected drop in precision reflected the natural class imbalance in systematic review, where eligible articles comprise a small fraction of search results. For reference, performance metrics were calculated from the original Cochrane Library reviews using their reported numbers of included and excluded articles after abstract screening.^17–39^ While LLM precision (range 0,004-0.096) was lower than the Cochrane reviewers’ (0.235), several models maintained high sensitivity: GPT-3.5 (1.000, GPT-4o (0.904), Llama 3 (0.841), and Sonnet 3.5 (0.823). Consequently, LLM screening exhibited potential for reducing researcher workload by automated exclusion of ineligible records.

**Table 3.** Performance of 5 LLMs used with their respective optimal prompts across the comprehensive dataset (n = 119 695), compared to an expected performance ceiling calculated from the reported number of included abstracts in the original Cochrane systematic reviews used for experiments.

Human/Model	Optimal prompt	Sensitivity (recall)	Specificity	Balanced accuracy	Precision (PPV)	NPV	F1-score
Cochrane	N/A	1.000	0.993	0.996	0.235	1.000	0.381
GPT-3.5	Heavy	1.000	0.419	0.710	0.004	1.000	0.008
GPT-4o	Extreme	0.904	0.949	0.926	0.038	1.000	0.074
Gemini 1.5 Pro	Heavy	0.756	0.976	0.866	0.068	0.999	0.125
LLaMA 3	None	0.841	0.776	0.809	0.008	1.000	0.017
Sonnet 3.5	None	0.823	0.982	0.903	0.096	1.000	0.172

Performance calculated from Cochrane Library review data was superior to all tested LLMs. Precision was expectedly low for all screeners, likely driven by a low prevalence of eligible articles for inclusion. N/A = not applicable.

### Ensemble configurations exhibit useful abstract screening performance

**Figure 3.**
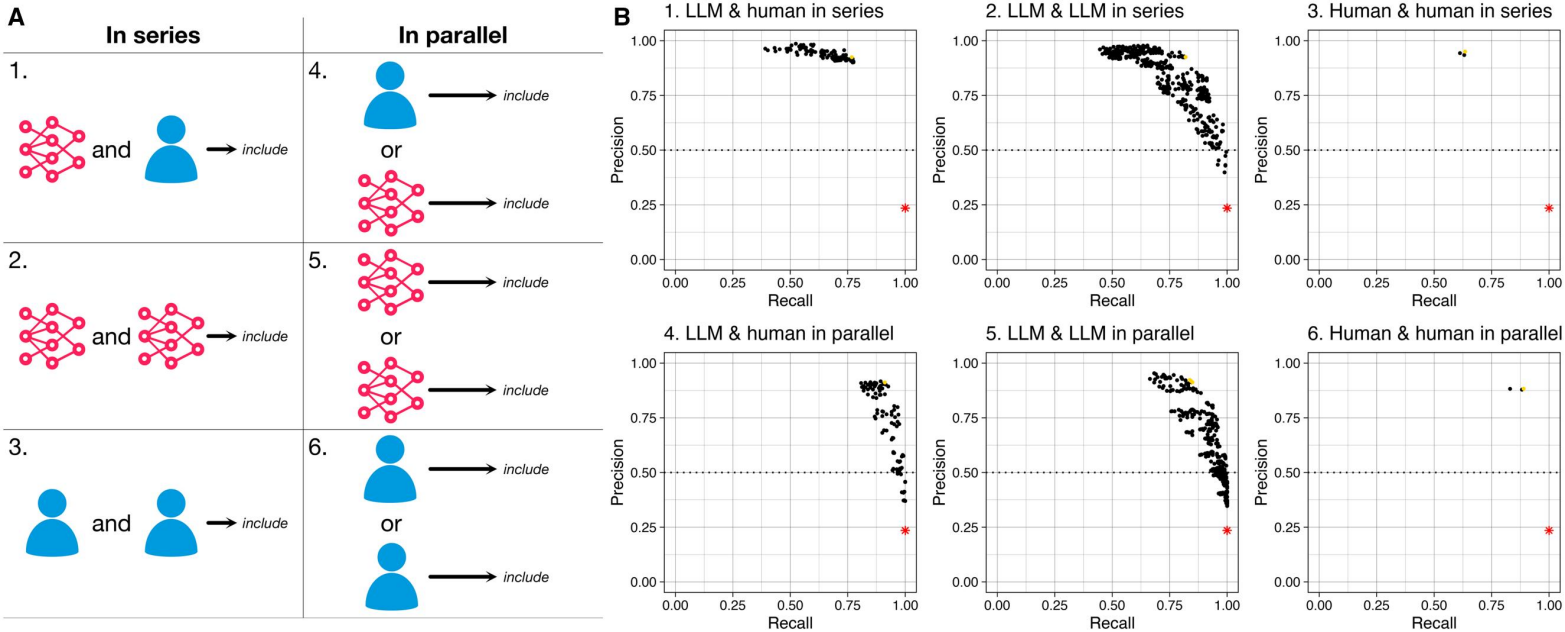
(A) Schematics describing 6 distinct configurations for incorporation of LLM and human decisions into a binary ensemble system. (B) Precision (positive predictive value) and recall (sensitivity) of every ensemble permutation combining LLMs, prompts, and human researchers in each configuration; tested across the development dataset (n = 800). For each configuration, the ensemble with the highest calculated accuracy is colored gold. For comparison, calculated performance from the original Cochrane reviews is indicated with red asterisks. Sixty-six ensembles across 2 parallel configurations obtained maximal sensitivity, all with higher precision than the Cochrane reviewers (albeit across the balanced dataset). GPT-3.5 with the “heavy” bias prompt exhibited the highest precision (0.458) while maintaining 100% sensitivity in 10 combinations. While many LLM-LLM in series ensembles approached perfect sensitivity, the best performing system was GPT-3.5 with “heavy” bias prompt and Sonnet with “extreme” bias prompt (sensitivity = 0.996).

LLMs and human researcher decisions—from experiments involving the development dataset—were combined in series and in parallel, in 6 distinct configurations (Figure 3). Combination in series meant that both component decisions had to be “include” for articles to be included; combination in parallel meant that only one “include” decision was required for inclusion. As predicted, series ensembles exhibited greater average precision, while parallel ensembles exhibited greater sensitivity as they had a lower barrier to inclusion. Moreover, 66 ensembles belonging to 2 parallel configuration schema exhibited perfect sensitivity: LLM and human in parallel, and LLM and LLM in parallel. Every ensemble with perfect sensitivity had a higher precision than recorded by the original Cochrane reviewers (Figure 3). Many LLM-LLM ensembles approached perfect sensitivity, the closest being GPT-3.5 with “heavy” bias prompt and Sonnet with “extreme” bias prompt (sensitivity = 0.996).

The highest precision of an LLM-LLM ensemble with perfect sensitivity was 0.458, exhibited by GPT-3.5 with “heavy” bias prompt combined with any of the following models in parallel: Sonnet with “none” bias prompt, GPT-4 with “none” bias prompt, GPT-4 with “mild” bias prompt, GPT-4 with “moderate” bias prompt, GPT-4 with “heavy” bias prompt, GPT-4o with “none” bias prompt, GPT-4o with “mild” bias prompt, GPT-4o with “moderate” bias prompt, GPT-4o with “heavy” bias prompt, and Gemini Pro with “none” bias prompt. One LLM-human ensemble attained the same precision of 0.458 with perfect sensitivity: GPT-3.5 with “heavy” bias and Bravo. Six LLM-human ensembles and 60 LLM-LLM ensembles attained perfect sensitivity in total.

LLM-human ensemble evaluation was limited to the development dataset due to the infeasibility of independently replicating human screening across the comprehensive dataset. As seen with results from individual LLMs, ensemble precision tended to drop as the number of ineligible articles to screen is increased. When tested over the comprehensive dataset, LLM-LLM ensembles exhibited precision scores between 0.0036 and 0.1450 (Table 4), frequently higher than any individual LLM (Table 3). Four parallel ensembles exhibited 100% sensitivity, a prerequisite for autonomous deployment: GPT-3.5 with “heavy” prompt combined with GPT-4o with “extreme” prompt, Gemini 1.5 Pro with “heavy” prompt, Llama 3 with “none” prompt, or Sonnet 3.5 with “none” prompt. Of these perfect-sensitivity ensembles, the maximal workload reduction was 41.81%, calculated as the proportion of screened articles that were correctly excluded. The highest measured workload reduction was 99.13%—by Gemini 1.5 Pro (“heavy” bias) and Sonnet 3.5 (“none” bias) in series—but this was at the expense of a lower sensitivity of 69%.

**Table 4.** Optimal LLM-LLM ensemble performance across the comprehensive dataset (n = 119 695).

Model 1	Model 2	Configuration	Sensitivity (Recall)	Specificity	Balanced Accuracy	Precision (PPV)	NPV	F1-score	Workload reduction
GPT-4o (extreme)	GPT-3.5 (heavy)	Parallel	1.0000	0.4173	0.7087	0.0039	1.0000	0.0077	41.64%
GPT-4o (extreme)	Gemini 1.5 Pro (heavy)	Parallel	0.9336	0.9409	0.9373	0.0346	0.9998	0.0668	93.88%
GPT-4o (extreme)	Llama 3 (none)	Parallel	0.9520	0.7623	0.8572	0.0090	0.9999	0.0178	76.06%
GPT-4o (extreme)	Sonnet 3.5 (none)	Parallel	0.9373	0.9453	0.9413	0.0374	0.9998	0.0720	94.31%
GPT-3.5 (heavy)	Gemini 1.5 Pro (heavy)	Parallel	1.0000	0.4179	0.7090	0.0039	1.0000	0.0077	41.70%
GPT-3.5 (heavy)	Llama 3 (none)	Parallel	1.0000	0.3764	0.6882	0.0036	1.0000	0.0072	37.55%
GPT-3.5 (heavy)	Sonnet 3.5 (none)	Parallel	1.0000	0.4190	0.7095	0.0039	1.0000	0.0078	41.81%
Gemini 1.5 Pro (heavy)	Llama 3 (none)	Parallel	0.9151	0.7703	0.8427	0.0090	0.9998	0.0177	76.86%
Gemini 1.5 Pro (heavy)	Sonnet 3.5 (none)	Parallel	0.8893	0.9681	0.9287	0.0596	0.9997	0.1116	96.59%
Llama 3 (none)	Sonnet 3.5 (none)	Parallel	0.9151	0.7734	0.8443	0.0091	0.9998	0.0180	77.17%
GPT-4o (extreme)	GPT-3.5 (heavy)	Series	0.9041	0.9507	0.9274	0.0399	0.9998	0.0765	95.11%
GPT-4o (extreme)	Gemini 1.5 Pro (heavy)	Series	0.7269	0.9841	0.8555	0.0943	0.9994	0.1669	98.46%
GPT-4o (extreme)	Llama 3 (none)	Series	0.7934	0.9623	0.8778	0.0456	0.9995	0.0863	96.28%
GPT-4o (extreme)	Sonnet 3.5 (none)	Series	0.7897	0.9857	0.8877	0.1117	0.9995	0.1957	98.62%
GPT-3.5 (heavy)	Gemini 1.5 Pro (heavy)	Series	0.7565	0.9779	0.8672	0.0722	0.9994	0.1317	97.84%
GPT-3.5 (heavy)	Llama 3 (none)	Series	0.8413	0.8190	0.8302	0.0104	0.9996	0.0206	81.94%
GPT-3.5 (heavy)	Sonnet 3.5 (none)	Series	0.8229	0.9827	0.9028	0.0976	0.9996	0.1746	98.32%
Gemini 1.5 Pro (heavy)	Llama 3 (none)	Series	0.6827	0.9822	0.8324	0.0800	0.9993	0.1432	98.27%
Gemini 1.5 Pro (heavy)	Sonnet 3.5 (none)	Series	0.6900	0.9908	0.8404	0.1450	0.9993	0.2396	99.13%
Llama 3 (none)	Sonnet 3.5 (none)	Series	0.7491	0.9850	0.8671	0.1020	0.9994	0.1796	98.55%

Multiple parallel ensembles approached or achieved perfect sensitivity. Precision was lower across the comprehensive dataset than the development dataset, as expected due to data imbalance (relatively few articles eligible for inclusion). However, due to the overrepresentation of ineligible articles in real-world abstract screening, LLMs confer substantial potential efficiency gains. The maximal workload reduction (proportion of articles correctly excluded) was 99.13% overall, and 41.81% for ensembles that exhibited perfect sensitivity.

## Discussion

Optimal combinations of LLMs and prompts—as individual models or in ensembles—can exhibit perfect sensitivity (recall) and sufficient precision (positive predictive value) to reduce the abstract screening workload in systematic review. LLM precision dropped when extended to the comprehensive dataset because relatively few screened articles are eligible for inclusion; similarly, Cochrane author precision was lower than researchers replicating screening over a balanced subset of the retrieved records. However, because of the overrepresentation of ineligible articles during abstract screening, LLMs can reduce workload significantly. Here, a maximal workload reduction of 41.81% was exhibited by an ensemble with perfect sensitivity. Performance variation between different models and prompts illustrates the importance of LLM selection, prompt engineering, and domain-specific validation when deploying LLMs for autonomous abstract screening. Performance variation between reviews highlights the importance of factors such as clarity of inclusion criteria and reporting to ensure LLM screening is optimized and reproducible.^6^^,^^42^

The relative performance of LLMs may be more favorable than comparisons to the original reviews suggest. The performance ceiling calculated from the original reviews was likely inflated by use of review authors’ decisions to define the ground truth, authors’ subject matter expertise and preconceived notions of what types of study were supposed to be included, as well as mistakes or omissions in descriptions of the search and screening process. When formally tested, trained human researchers exhibit a lower abstract screening sensitivity than the performance ceiling calculated here from Cochrane review data: 87%, improving to 97% when 2 human researchers screen each abstract.^43^ LLMs exceeded this benchmark and may therefore complement conventional screening and reduce the workload for human researchers. Moreover, LLM abstract screening may improve the quality of evidence synthesis by reducing the number of eligible records that are lost.

Previous proof-of-concept studies have evaluated LLM abstract screening but have been highly restricted in terms of subject matter or failed to provide comparators to contextualize results.^10^^,^^44–46^ Various other approaches to automation have also been tested, including conventional machine learning techniques.^47^ Here, a prompt engineering strategy for automated abstract screening worked well with a wide variety of LLMs, albeit with variable performance between reviews. Potential applications could change the methodology of systematic review by working in series or in parallel with human researchers. With sufficient sensitivity demonstrated over a subset of studies, models could be entrusted with autonomously pre-screening studies to reduce the number of studies requiring human evaluation: working in series to maximize efficiency of screening, with the risk of erroneously excluding eligible studies that cannot be salvaged. Automated systems may instead be used in place of a second reviewer in parallel with human researchers. This would halve the initial screening workload by operating across the whole number of identified records, potentially capturing mistakenly excluded and included studies to improve screening accuracy and reduce the burden of full-text screening. For models designed to work in parallel, sensitivity may be sacrificed to maximize accuracy and thereby efficiency as more ineligible records can be excluded before full text screening. Specific fine-tuning may be employed to optimize performance and model behavior, although careful validation is required as customized models do not necessarily exhibit superior performance.^48^ Alternatively, rather than binary output to determine whether records should be included or excluded, a combination of prompt engineering and fine-tuning could be employed to generate uncertainty estimates which could guide human researchers to review records where model decisions are less likely to be accurate.^49^

Three limitations may have affected the study’s results and conclusions. First, representativeness was limited although a full issue of the *Cochrane Database of Systematic Reviews* was used to test across a broad range of medical topics. Inter-review variation shows that automated screening may be better suited to some subjects than others, and applications should be specifically validated within a subject or topic if used. Further work may seek to explore where LLM screening is most effective, and how review protocols and screening criteria could be better designed to facilitate automation. LLMs could even be used as a tool to quantify the clarity and reproducibility of screening described in systematic review reports. Second, the study may have exhibited an optimization bias in favor of GPT-3.5 as initial prompt engineering was undertaken using that LLM in smaller scale experiments. This was due to relative ease and lower cost of access. Further improvement in the performance of each LLM is likely feasible with more intensive prompt engineering, which could be specifically directed to the aims of a single review.^12^ Finally, the performance of Cochrane reviewers were likely inflated by their use as both a comparator and as ground truth, as well as due to any mistakes, omissions, or unclearly communicated aspects in the reviewers’ search and screening strategies.^40^ While the performance of the original reviewers serves as a useful benchmark corresponding to maximal possible accuracy, the alternative comparator provided by independent researchers replicating screening is a more useful gauge of the relative strengths and limitations of LLM-based screening and also lay closer to previous estimates of human screening performance.^43^

Further work is required to integrate automated abstract screening into the conventional workflow of systematic review: our approach requires accessing an API with a spreadsheet containing details from every study identified at the search stage. By providing comprehensive detail about the LLMs used and our broader methodology, we aim to maximize reproducibility of our results and access to automated abstract screening.^50^ However, code-free solutions would enable more researchers to leverage automated abstract screening in their research.^51^ The institution of EBM relies heavily upon accurate syntheses of available evidence to answer clinical questions, of which systematic review forms a critical component. It is therefore critical that the implementation of automated abstract screening does not compromise the quality or reproducibility of systematic review.^11^ We would recommend authors report any use of automated screening technology clearly enough for other researchers to replicate their approach, including details about the model and prompt used, and how automated screening contributed to inclusion decisions. Ideally, the Preferred Reporting Items for Systematic Reviews and Meta-Analyses (PRISMA) should include these details as automated screening becomes common practice.^5^

## Conclusion

LLMs can facilitate automated abstract screening with high sensitivity, best operating in parallel with human researchers. Automated abstract screening may improve the efficiency and quality of systematic review and could thereby improve the practice of EBM. LLM performance is subject-specific but can be optimized through prompt engineering, and researchers are advised to conduct domain-specific validation before unsupervised deployment.

## Supplementary Material

ocaf050_Supplementary_Data

## Data Availability

All study data are available in the study supplement.
