# Supplementary material for: High-performance automated abstract screening with large language model ensembles
Source: J Am Med Inform Assoc. 2025 Mar 22;32(5):893–904. doi: 10.1093/jamia/ocaf050 (PMC12012331; doi:10.1093/jamia/ocaf050)
Supplement: ocaf050_Supplementary_Data [file ocaf050_supplementary_data.zip › Supplementary Files/Supplementary Material 1.rtf]

Bias Level: NoneYou are screening articles for inclusion in the systematic review titled ‘{SR_title}’. Using the inclusion criteria for the systematic review provided below, decide if the screened articles should be included based on their title and abstract. Respond only with either ‘Include’ or ‘Exclude’.	Systematic Review Title: {SR_title}	Inclusion Criteria (PICOS): {incl_criteria}	Exclusion Criteria: {excl_criteria}	Article Title: {title}	Article Abstract: {abstract}Bias Level: MildYou are screening articles for inclusion in the systematic review titled ‘{SR_title}’. Using the inclusion criteria for the systematic review provided below, decide if the screened articles should be included based on their title and abstract. Where relevance to the systematic review title is apparent, and the article meets most inclusion criteria, favour inclusion unless clear exclusion criteria are met. Respond only with either ‘Include’ or ‘Exclude’.	Systematic Review Title: {SR_title}	Inclusion Criteria (PICOS): {incl_criteria}	Exclusion Criteria: {excl_criteria}	Article Title: {title}	Article Abstract: {abstract}Bias Level: ModerateYou are screening articles for inclusion in the systematic review titled ‘{SR_title}’. Using the inclusion criteria for the systematic review provided below, decide if the screened articles should be included based on their title and abstract. If the article seems likely to be relevant to the systematic review’s theme, and it does not explicitly meet any exclusion criteria, lean towards inclusion. Respond only with either ‘Include’ or ‘Exclude’.	Systematic Review Title: {SR_title}	Inclusion Criteria (PICOS): {incl_criteria}	Exclusion Criteria: {excl_criteria}	Article Title: {title}	Article Abstract: {abstract}Bias Level: HeavyYou are screening articles for inclusion in the systematic review titled ‘{SR_title}’. Using the inclusion criteria for the systematic review provided below, decide if the screened articles should be included based on their title and abstract. If there is any uncertainty if an article fulfils the inclusion criteria, but the abstract appears relevant to the title of the systematic review, lean towards including the article, as long as no exclusion criteria are met. Respond only with either ‘Include’ or ‘Exclude’.	Systematic Review Title: {SR_title}	Inclusion Criteria (PICOS): {incl_criteria}	Exclusion Criteria: {excl_criteria}	Article Title: {title}	Article Abstract: {abstract}Bias Level: ExtremeYou are screening articles for inclusion in the systematic review titled ‘{SR_title}’, with heavy preference towards inclusion. Your primary goal is to include articles unless there are overwhelming reasons to exclude. Apply the inclusion criteria very loosely and the exclusion criteria extremely strictly. Assume relevance unless proven otherwise beyond any reasonable doubt. Prioritise maximum sensitivity. Exclude only if the article is unequivocally irrelevant or explicitly violates multiple exclusion criteria. Respond only with ‘Include’ or ‘Exclude’.	Systematic Review Title: {SR_title}	Inclusion Criteria: {incl_criteria}	Exclusion Criteria: {excl_criteria}	Article Title: {title}	Abstract: {abstract}Bias Level: TitleYou are screening articles for inclusion in the systematic review titled ‘{SR_title}’. Using the systematic review title, decide if the screened articles should be included based on the relevance of their title and abstract. If there is any uncertainty, lean towards including the article. Respond only with either ‘Include’ or ‘Exclude’.	Systematic Review Title: {SR_title}	Article Title: {title}	Article Abstract: {abstract}
